# A Snapshot of Early Transcriptional Changes Accompanying the Pro-Neural Phenotype Switch by NGN2, ASCL1, SOX2, and MSI1 in Human Fibroblasts: An RNA-Seq Study

**DOI:** 10.3390/ijms252212385

**Published:** 2024-11-18

**Authors:** Ekaterina M. Samoilova, Daria A. Chudakova, Erdem B. Dashinimaev, Anastasiya V. Snezhkina, Olga M. Kudryashova, Anastasia V. Lipatova, Alesya V. Soboleva, Pavel O. Vorob’yev, Vladimir T. Valuev-Elliston, Natalia F. Zakirova, Alexander V. Ivanov, Vladimir P. Baklaushev

**Affiliations:** 1 Federal Center for Brain and Neurotechnologies, Federal Medical and Biological Agency of Russia, 117513 Moscow, Russia; samoyket@gmail.com (E.M.S.); olga.kudryashova179@gmail.com (O.M.K.); asobolforw@gmail.com (A.V.S.); 2 Engelhardt Institute of Molecular Biology, Russian Academy of Sciences, 119991 Moscow, Russia; leftger@rambler.ru (A.V.S.); lipatovaanv@gmail.com (A.V.L.); pavel.gealbhain@gmail.com (P.O.V.); gansfaust@mail.ru (V.T.V.-E.); aivanov@yandex.ru (A.V.I.); 3 National Medical Research Center of Children’s Health of the Ministry of Health of the Russian Federation, 119296 Moscow, Russia; 4 Center for Precision Genome Editing and Genetic Technologies for Biomedicine, Pirogov Russian National Research Medical University, 117997 Moscow, Russia; dashinimaev@gmail.com; 5 Federal Research and Clinical Center of Specialized Medical Care and Medical Technologies Federal Medical and Biological Agency of Russia, 115682 Moscow, Russia; 6 Pulmonology Research Institute, Federal Medical and Biological Agency of Russia, 115682 Moscow, Russia; 7 Department of Medical Nanobiotechnology of Medical and Biological Faculty, Pirogov Russian National Research Medical University, Ministry of Health of the Russian Federation, 117997 Moscow, Russia

**Keywords:** cell fate, cell therapy, cell reprogramming, RNA-seq

## Abstract

Direct pro-neural reprogramming is a conversion of differentiated somatic cells to neural cells without an intermediate pluripotency stage. It is usually achieved via ectopic expression (EE) of certain transcription factors (TFs) or other reprogramming factors (RFs). Determining the transcriptional changes (TCs) caused by particular RFs in a given cell line enables an informed approach to reprogramming initiation. Here, we characterized TCs in the human fibroblast cell line LF1 on the 5th day after EE of the single well-known pro-neural RFs NGN2, ASCL1, SOX2, and MSI1. As assessed by expression analysis of the bona fide neuronal markers nestin and beta-III tubulin, all four RFs initiated pro-neuronal phenotype conversion; analysis by RNA-seq revealed striking differences in the resulting TCs, although some pathways were overlapping. ASCL1 and SOX2 were not sufficient to induce significant pro-neural phenotype switches using our EE system. NGN2 induced TCs indicative of cell phenotype changes towards neural crest cells, neural stem cells, mature neurons, as well as radial glia, astrocytes, and oligodendrocyte precursors and their mature forms. MSI1 mainly induced a switch towards early stem-like cells, such as radial glia.

## 1. Introduction

One of the most difficult tasks of modern translational medicine is the replenishment of the cells that are lost in the central nervous system (CNS) as a result of injury or disease. First and foremost, this is due to the fact that in adult humans, the regenerative potential of the CNS is staggeringly low (or absent), and approaches have not yet been established to evoke its intrinsic regenerative ability. Thus, cell therapy must “come to the aid” of doctors, in the form of either transplantation of pro-neural/neural cells or in situ pro-neural reprogramming at the lesion site to compensate for the cell loss. Furthermore, there are two possible sources of neural cells in case of transplantation therapy, i.e., either direct reprogramming (conversion of any type of differentiated somatic cells to neuronal cells without an intermediate pluripotency stage) or reprogramming through the induced pluripotent stem cell (iPSC) step. Both methods have their limitations, advantages, and disadvantages, comprehensively described elsewhere. One of the disadvantages of direct reprogramming is the lack of standard protocols allowing for a reproducible cell type conversion to the particular chosen phenotype in a given condition [1]. Most protocols of direct reprogramming are based on the constitutive or transient ectopic expression (EE) of certain transcription factors (TFs) or regulatory proteins (regulatory factors, RFs). Fibroblasts or mononuclear blood cells are abundant and can be obtained via a relatively non-invasive procedure; thus, the majority of works in the field of reprogramming were performed on these two types of cells, with the ultimate goal of using them for autologous transplantation.

Previously, it was demonstrated in mouse fibroblasts that each pair of TFs used for reprogramming towards cells with neuronal features evokes unique TCs [2]. Undoubtedly, determining specific transcriptional signature(s) for particular RFs in human cells will enable the subsequent generation of cells with known patterns of gene expression and, hence, the chosen phenotype and functional properties. A comparison of such TCs might also help to define a common (“core”) transcriptional signature associated with cell fate switch towards the neuronal lineage, as well as key pathways involved. In turn, the pharmacological modulation of such pathways might be utilized to enhance RF-induced reprogramming or used for chemical reprogramming without any RFs. Transcriptomic data can also be used to find novel RFs with modes of action similar to those of known ones. Indeed, multi-omics data, including transcriptomics, have been previously used as a foundation for the data-driven prediction of novel RFs [3]. Further, although in the aforementioned Tsunemoto’s work TFs were used in pairs, perhaps single TFs are also sufficient to induce reprogramming [2]. For example, it has been claimed that a single TF, ASCL1, can be used to reprogram human fibroblasts into the so-called induced neurons (iNs) [4]. NGN2 in combination with Brn3a was sufficient to induce fibroblasts conversion to sensory neurons [5], and singly expressed SOX2 was sufficient to convert NG2 glia cells to neurons [6].

From all the variety of RFs, ASCL1, NGN2, and MSI1 are the ones that have already been used successfully for pro-neural reprogramming [5,7], often in combination with other factors and/or small molecules. ASCL1 and NGN2 are so-called “pioneer” TFs, capable of binding heterochromatin, re-shaping it to make it transcription-permissive, and further recruiting so-called “cooperative” TFs to initiate transcriptional cascades [8]. They are necessary for the induction of reprogramming towards the neural lineage (comprehensively reviewed in [9]). Also, “cocktails” of pro-neural reprogramming TFs might include TFs that induce transcriptional signatures of pluripotency, such as SOX2 [10,11,12,13,14,15], and the addition of pro-neural TFs and subsequent cell cultivation in a medium supplemented with neuronal growth factors might be sufficient for a corresponding cell fate change. For example, ASCL1 and NGN2 were used in combination with SOX2 to convert mesenchymal stem cells to mature neurons [16]. The exact contribution of SOX2 to this process is yet to be defined. NGN2 was used in several works to convert human fibroblasts into neurons, as a single reprogramming factor or in combination with other TFs (comprehensively reviewed in [17]). Finally, MSI1 is a neural RNA-binding protein and a marker of neural progenitor cells (including neural stem cells) [18], ensuring a proliferative stem-like state in neural progenitors. As a component of the “cocktail of reprogramming factors”, MSI1, alongside NGN2 and MBD2, was used to generate functional neural precursor cells from non-neuronal somatic cells [19]; however, the question remains of whether it can induce the same cell fate switch if used without NGN2 and MBD2.

Despite the omnium gatherum of different reprogramming protocols used in a significant number of works (including the aforementioned ones) and supposedly leading to pro-neural cell conversion, there is still a gap of knowledge in the field with several unanswered questions resulting from limitations of the majority of such studies. The limitations are the simultaneous use of different RFs rather than single ones, the assessment of TCs in late stages of reprogramming rather than in the early stage, which determines the reprogramming trajectory, the lack of a side-by-side comparison of different single RFs, a tendency to use rodent-derived cell lines rather than human cells, and the use of different reprogramming tools (the RFs being delivered to the cells by different vectors), which makes such studies incomparable and non-combinable. The still unanswered questions are as follows.

Firstly, what are the earliest TCs initiating such conversion, the ones that are the very cause rather than just a consequence of the pro-neural cell phenotype switch? Unless such TCs are determined, no informed approach to the modulation of cell fate conversion via tools other than RFs (such as small molecules, microRNAs, etc.) can be taken. However, only a few works focused on TCs in the earliest steps of the initiation of the pro-neuronal cell phenotype shift in fibroblasts. Of them, only a few were performed in human cells. Most of them used combinations of RFs rather than single RFs (thus, making it impossible to assess the impact of individual RFs), and none of them determined at a chosen time point both the TCs kindled by RFs and the whole spectrum of pro-neuronal phenotypes potentially arising as a result of such EE, as suggested by the bioinformatic analysis of the transcriptome. At the same time, the assessment of the “trajectory” of the pro-neural phenotype switch based on the patterns of expression of only a few neuronal markers, done in many works, does not provide a whole picture of the cell conversion trajectories and might result in “false positive” results if the expression of such markers is elevated in the absence of other TCs necessary for the pro-neuronal cell fate shift.

Secondly, are there common “master” pathways targeted by functionally different pro-neural RFs, assessed based on these TCs? Identifying such pathways is key to the focused modulation of the pro-neural phenotype change.

There is also no published side-by-side comparison of TCs and associated changes in the composition of the resulting cell subpopulations caused by different single RFs in the same cells at the same time point using the same construct to deliver the reprogramming transgene. Our work might be a first step towards the creation of a shared library of such standardized data to be used (and expanded) by anyone working in the field. Thus, we chose the most commonly used cells and widely available tools for transgene delivery.

To address all of this, here we determined and compared the early TCs evoked by the ectopic expression of single ASCL1, SOX2, NGN2, and MSI1 in the human embryonic lung fibroblast cell line LF1, not necessarily resulting in direct reprogramming, but shifting the cells towards pro-neural phenotype acquisition.

The subsequent analysis revealed diverse transcriptome trajectories driving phenotype conversion, the transcription signatures of a variety of resulting cell subpopulations, as well as the main pathways involved. The transcriptome datasets generated in this article can be combined with other datasets and analyzed independently using other bioinformatic approaches, thus laying a foundation for multiple future studies.

## 2. Results

### 2.1. The Single Ectopic Expression of ASCL1, SOX2, NGN2, and MSI1 Evokes the Expression of Nestin and β-III-Tubulin in LF1 Cells

On the 5th day, significantly elevated mRNA levels of ASCL1, SOX2, NGN2, and MSI1 in the target cells were confirmed by qRT-PCR (Supplementary File S1 Figure S1), and the cells displayed visible morphological changes.

**Figure 1:**
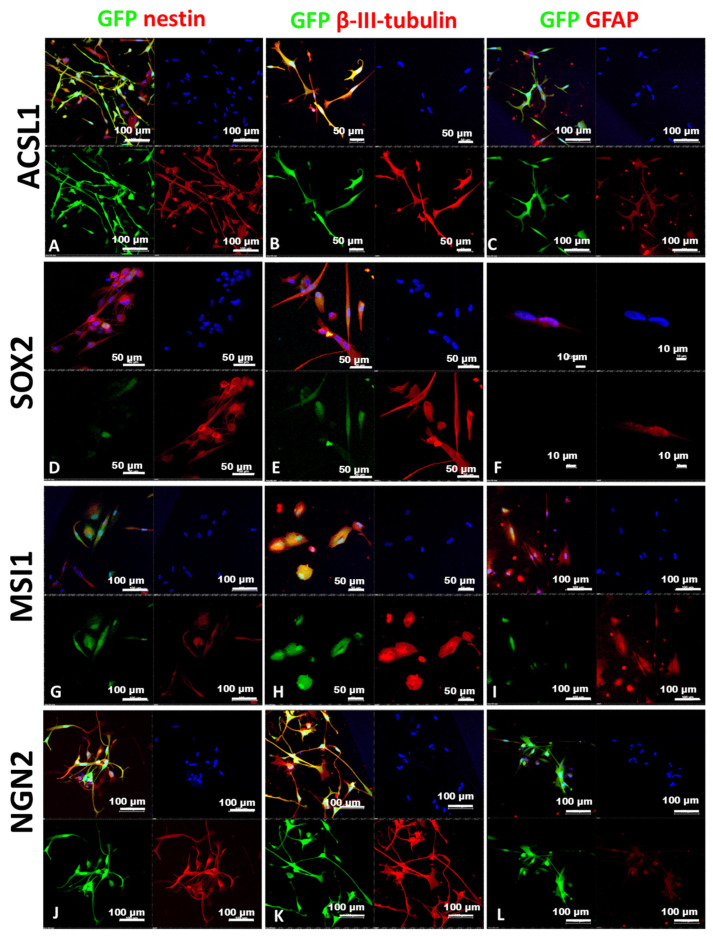
Immunocytochemical analysis of LF1 cells on day 5 after transduction. Confocal microscopy images of cells stained for nestin, β-III-tubulin, and GFAP. LF1-ASCL1 (**A**–**C**), LF1-SOX2 (**D**–**F**), LF1-MSI1 (**G**–**I**), LF1-NGN2 (**J**–**L**) samples. Green channel: GFP fluorescence confirming the integration of the lentiviral vectors. Red channel: fluorescence of the secondary antibodies (Alexa Fluor 633). The cell nuclei are stained with Hoechst (blue) in all the panels.

The protein levels of nestin and β-III-tubulin (bona fide markers of the pro-neural phenotype switch, most often used in similar studies to confirm the initiation of pro-neural reprogramming) were assessed by immunocytochemistry staining. The levels of GFAP (marker of astrocytic cells) were also assessed. In cells expressing ASCL1, SOX2, NGN2, and MSI1, there was a marked elevation in the levels of all these markers (Figure 1), which are commonly non-detectable in LF1 cells. The highest levels of expression of β-III-tubulin and nestin were observed in the samples LF1-NGN2 and LF1-MSI1 (LF1 cells transduced with LeGOiG2-Puro+NGN2 and LeGOiG2-Puro+MSI1, correspondingly) (Figure 1), which is not surprising, given the role of MSI1 in neural stem cells [18,20,21]. Thus, we confirmed that each of the tested RFs shifted the cells to a “neuronal-like” phenotype.

### 2.2. RNA-Seq Data Analysis 

**Figure 2:**
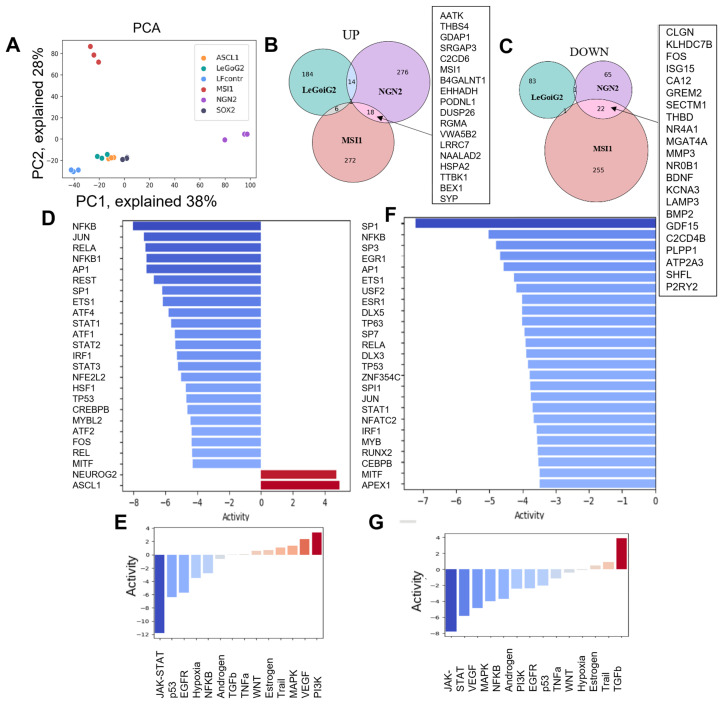
Differential expression analysis of RNA-seq data (**A**) Principal component analysis (PCA) of the log2 RNA-seq data presented as a two-dimensional scatterplot of the first two principal components. The sample groups are represented by different colors, each dot represents a biological replicate; the sample legend is provided within the plot; (**B**,**C**) Venn diagrams of the DEGs across LF1-NGN2, LF1-MSI1, and LF1-LeGoiG2 that were up- (**B**) and down- (**C**) regulated. (**D**–**G**) For LF1-NGN2 and LF1-MSI1, based on each TF’s targets amongst the DEGs, the top 25 most highly affected TFs are shown ((**D**,**F**), correspondingly), as well as the estimated pathway activity ((**E**,**G**), correspondingly).

Bulk RNA-seq data analysis was performed as described, and lists of the differentially expressed genes (DEGs) were generated for each type of sample (Supplementary File S2). Principal component analysis (PCA) of RNA-seq data showed that the samples LF1-MSI1 and LF1-NGN2 were distant from the LF1 and LF1-LeGoiG2-Puro+ controls (Figure 2A), whereas LF1-ASCL1 and LF1-SOX2 clustered near the LF1-LeGoiG2-Puro+ control (Figure 2A), which indicated a significant contribution of the empty lentiviral vector to the transcriptional changes in the cell. There was a partial overlap of the DEGs in the LF1-MSI1 and LF1-NGN2 samples (common 22 downregulated and 18 upregulated genes (Figure 2B,C, Supplementary File S1 Figure S2)). The gene expression patterns in the LF1-MSI1 and LF1-NGN2 samples suggested the activation and silencing of several signaling cascades that are not neuronal-exclusive (Figure 2E,G).

Next, we estimated transcription factor (TF) score activity based on each TF’s gene targets transcriptome data (Figure 2D,F). Among the top 25 TFs with the lowest activity scores, we found those common for LF1-NGN2 and LF1-MSI1: AP1, NFKB, JUN, TP53, STAT1. In the case of LF1-NGN2, FOS was also found among the TFs with the lowest activity scores, and ASCL1 and NEUROG2 among the most activated TFs.

### 2.3. Cellular Composition After Ectopic Expression of RFs, as Suggested by Bioinformatic Analysis: Stem-like and Neurogenic-like Subpopulations

**Figure 3:**
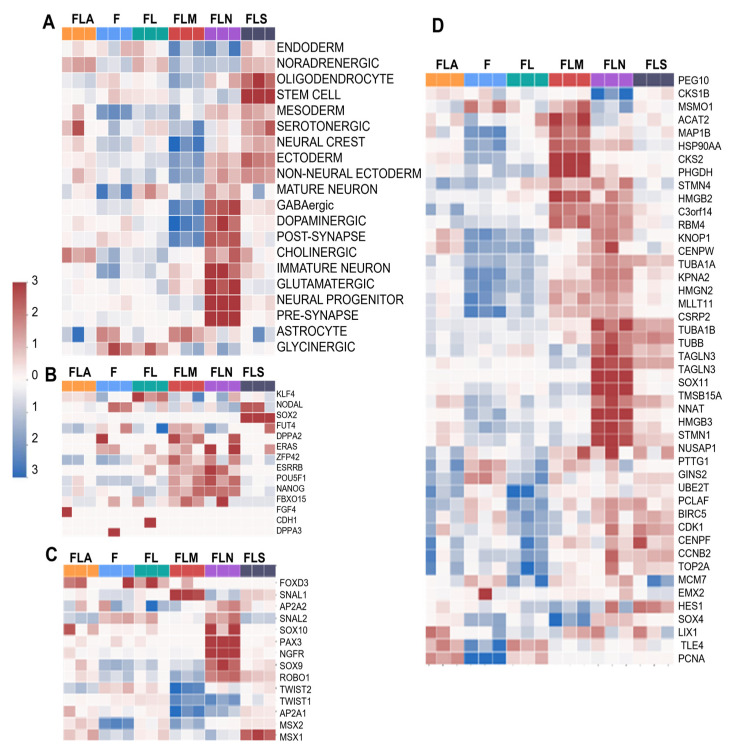
Profiles of cell subtypes in the samples based on gene expression analysis. Heatmap showing enrichment of the samples with DEGs of pro-neuronal cell populations (**A**), stem-like cells (**B**), neural crest cells (**C**), and radial glial cells (**D**). F, control fibroblasts LF1, FL, LF-LeGoiG2-Puro+, FLA, LF-LeGoiG2-Puro+-ASCL1, FLM, LF-LeGoiG2-Puro+-MSI1, FLN, LF-LeGoiG2-Puro+-NGN2, FLS, LF-LeGoiG2-Puro+-SOX2. Blue and red color scale represents the median-scaled change in gene expression or cell population enrichment (red—up, blue—down).

Based on cell population-specific gene sets from WikiPathways data, we estimated the single-sample Gene Set Enrichment Analysis (ssGSEA) score for each of the conditions and found that the LF1-NGN2 samples were enriched in populations with some transcriptional characteristics of neural progenitors, radial glia, precursors of oligodendrocytes, astrocytes, as well as pericytes (Figure 3A).

**Figure 4:**
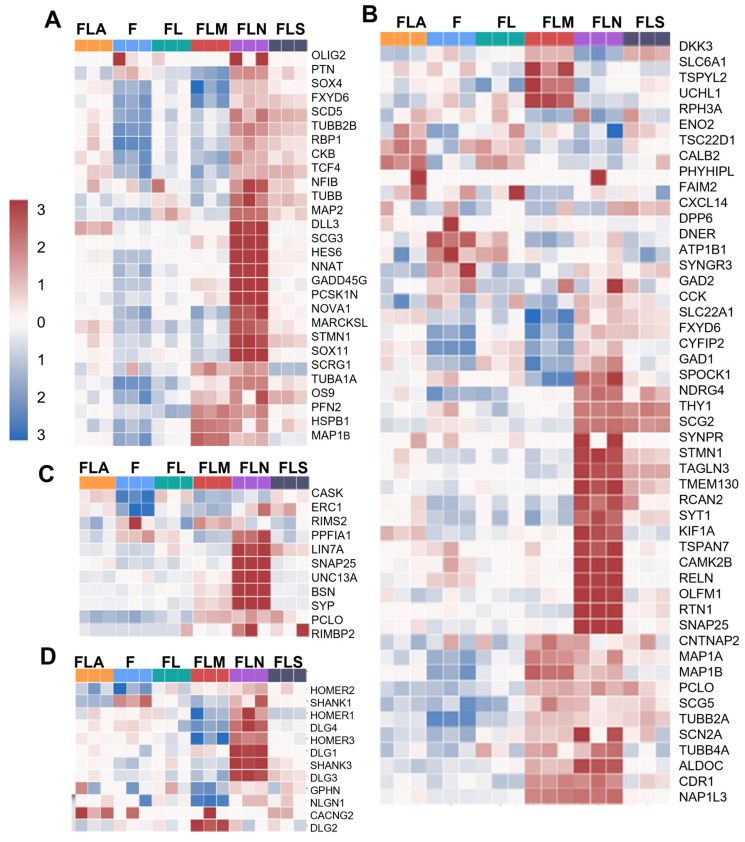
Profiles of cell subtypes in the samples based on gene expression analysis. Heatmap showing enrichment of the samples with DEGs of neural progenitors (**A**), neurons (**B**), presynaptic (**C**), and postsynaptic structures (**D**). F, control fibroblasts LF1, FL, LF-LeGoiG2-Puro+, FLA, LF-LeGoiG2-Puro+-ASCL1, FLM, LF-LeGoiG2-Puro+-MSI1, FLN, LF-LeGoiG2-Puro+-NGN2, FLS, LF-LeGoiG2-Puro+-SOX2. Blue and red color scale represents the median-scaled change in gene expression or cell population enrichment (red—up, blue—down).

The LF1-MSI1 sample was enriched with a stem cell population (Figure 3B). Surprisingly, EE indicated a contribution of NGN2 comparable to that of MSI1 to the stem cell population (Figure 3B). It also contributed to the formation of neural crest cells (Figure 3C) and radial glia (Figure 3D). The largest number of radial glia genes with elevated expression was in the samples LF1-NGN2 and LF1-MSI1, but the groups of upregulated genes in these samples had virtually no overlap (Figure 3D). Overall, in the LF1-NGN2 samples there was an upregulation of several neuron-associated genes such as *RBM4*, *TAGLN3*, *SOX11*, and *NNAT* (Figure 3D). According to GBMDeconvoluteR analysis, the LF1-NGN2 sample was the most enriched in neural progenitor cells among all samples (Figure 4A).

Populations of immature and mature neurons were present in the samples LF1-NGN2 and LF1-MSI1 (Figure 4B, Supplementary File S1 Figure S5). Moreover, the LF1-NGN2 samples were significantly more enriched in them than the LF1-MSI1 samples. The LF1-NGN2 samples were also enriched in genes which encode proteins important for presynaptic and postsynaptic structures (Figure 4C,D). The WikiPathways analysis revealed more “specialized” neuronal subtypes compared to GBMDeconvoluteR (Supplementary File S1 Figure S3). Subpopulations of immature neurons were identified in the samples LF1-NGN2 and LF1-MSI1 (Supplementary File S1 Figure S3B). The populations of GABAergic, dopaminergic, and cholinergic neurons were predominantly abundant in the LF1-NGN2 samples (Supplementary File S1 Figure S5C–E), whereas gene expression patterns common for glutamatergic neurons, as well as transcriptional signatures of serotonergic neurons, were found in both LF1-NGN2 and LF1-MSI1 (Supplementary File S1 Figure S3F).

### 2.4. Cellular Composition After Ectopic Expression of RFs, as Suggested by Bioinformatic Analysis: Glial Cells and Other Subpopulations

**Figure 5:**
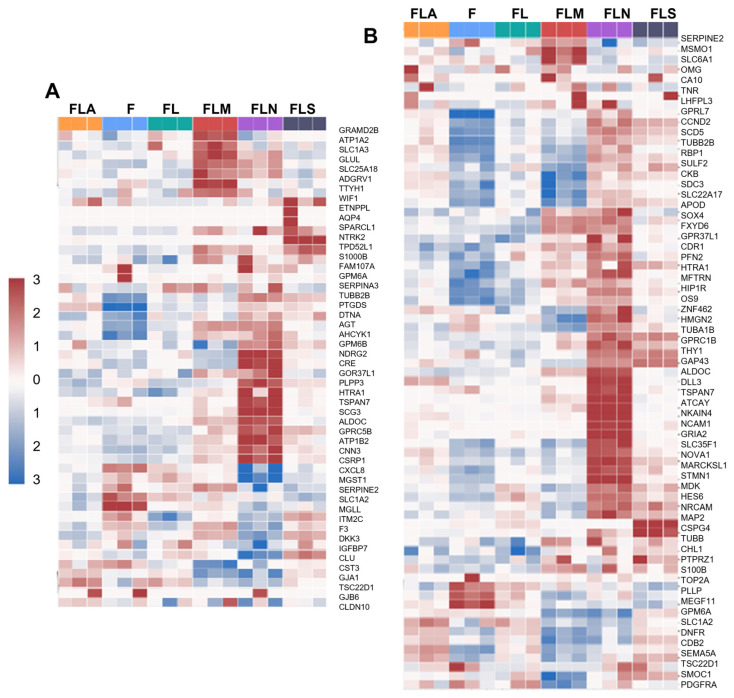
Profiles of cell subtypes in the samples based on gene expression analysis. Heatmap showing enrichment of the samples with DEGs of astrocytes (**A**) and oligodendrocyte precursors (**B**). F, control fibroblasts LF1, FL, LF-LeGoiG2-Puro+, FLA, LF-LeGoiG2-Puro+-ASCL1, FLM, LF-LeGoiG2-Puro+-MSI1, FLN, LF-LeGoiG2-Puro+-NGN2, FLS, LF-LeGoiG2-Puro+-SOX2. Blue and red color scale represents the median-scaled change in gene expression or cell population enrichment (red—up, blue—down).

Transcriptional signatures of astrocytic precursors, astrocytes, oligodendrocytes, and microglia were enriched in some samples (Figure 5, Supplementary File S1 Figures S4 and S5).

In particular, enrichment in gene sets determining the oligodendrocyte and oligodendrocyte precursor (OP) cell phenotype was observed predominantly in LF1-NGN2 (Figure 5B), and the number of the latter genes was higher than that of the former. The presence of astrocytic precursors (Supplementary File S1 Figure S4) and astrocytes (Figure 5A) might also be suggested. Of the so-called astrocytic genes, only three genes were upregulated in LF1-MSI1, namely, *S100B*, *METRN* (the gene that regulates glial cell differentiation and axon formation during neurogenesis), and *AGT*. At the same time, in LF1-MSI1, the transcription of some of the astrocytic genes was downregulated, for example, *CST3*, *GJA1*, and *TSC22D1*. The higher enrichment in transcriptional signatures of astrocytes was observed in LF1-NGN2 (Figure 5A), which is somewhat contradictory to our qRT-PCR data (of note, such discrepancies are not uncommon [22,23], as they reflect differences between methods and method limitations, and reinforce the need for the validation of the results by several methodologically different approaches). Lastly, in all samples, there was a marginal upregulation of the genes known to be associated with the microglial phenotype. Of them, the highest number of upregulated genes was in LF1-NGN2 and LF1-MSI1 (Supplementary File S1 Figure S5). 

## 3. Discussion

Almost 40 years after the publication of the first seminal work on direct cell reprogramming [24], leading to an “expansion” of the studies in this field, the molecular mechanisms of pro-neural cell fate switch still attract a great interest in biomedical research. In this work, we determined and comprehensively characterized early TCs caused by EE of single RFs in human embryonic lung fibroblasts in the initial stage of pro-neuronal cell phenotype conversion. Although some researchers managed to assess TCs at the very beginning of the reprogramming, at the 72 h time point [25], and even earlier [26] in both mouse and human cells, works reporting the early pro-neural TCs are still scarce, and our study fills this knowledge gap.

TFs are capable of acting in synergy (including in synergy with RFs other than TFs). The combination of TFs in a “cocktail of reprogramming factors” determines the resulting TCs and downstream pathways affected. In addition to synergistic interactions, TFs can also compete with each other, which, in particular, has been demonstrated for two members of the same family of TFs, ASCL1 and NGN2 [25,27]. Thus, we studied the TCs induced by each singly expressed RF to characterize RF-associated unique and common features related to cell fate switch, as it is known that co-expression of reprogramming TFs with other RFs can drastically change their distribution at targets (as it has been recently demonstrated for NGN2, for example [28]).

The recent review by Horisawa et al. summarized a significant number of pro-neural reprogramming protocols using different TFs [29]. From the list of these TFs, four RFs were chosen for our work, each representing one particular “type” of RF. ASCL1 is an “on-target” pioneer TF, capable of reprogramming fibroblasts to iNs, acting as a single RF [4]. The same is true for NGN2, another member of the same family of TFs [30] but a more “promiscuous” one, with a broad range of targets [31,32]. Despite being pro-neuronal TFs from the same family, the transcriptional signatures of ASCL1 and NGN2 only partially overlap, and in mice they induce the conversion of embryonic stem cells to neurons through different pathways [31]. SOX2 is a “stemness” TF that switches cells towards a more stem-like phenotype or the phenotype of neural stem cells [6]. MSI1 is an RF acting predominantly as a translation regulator [33], but it is known to affect the neuronal stem cell phenotype [34]. We assumed that if “master pathways” critical for pro-neuronal reprogramming exist, they will be common for all different RFs and could be identified by revealing the TC similarities in our samples.

One of the most commonly used approaches to deliver RFs to cells is via lentiviral vectors. Given that many works have utilized lentiviral constructs for cell reprogramming and keeping in mind that many subsequent works will be based on the usage of lentiviral constructs, we chose lentiviral vectors as a transgene delivery tool to make our data comparable and combinable with other data sets.

In our study, sets of genes upregulated by different RFs were almost non-overlapping, although 18 downregulated and 32 upregulated genes were in common between LF1-MSI1 and LF1-NGN2. Notably, in LF1-NGN2, not only upregulation, but also downregulation of a considerable number of genes was observed; these genes were predominantly the ones encoding proinflammatory proteins, chaperone proteins, tumor suppressors, and proteins involved in intercellular contacts. Among the common upregulated genes in LF1-NGN2 and LF1-MSI1, we found two genes related to TAU protein binding regulation: tau tubulin kinase 1 (*TTBK1*) and heat shock protein family A (*HSPA2*)-encoding genes. The proteins encoded by these genes regulate the dynamics of microtubules, which is essential for maintaining the structural integrity and proper functioning of neurons. Gene Set Enrichment Analysis (GSEA) for 22 common downregulated genes in LF1-NGN2 and LF1-MSI1 found enrichment in signaling receptor regulator activity, cytokine activity, and BMP receptor binding. Counterintuitively, among them, the gene *BDNF*, encoding brain-derived neurotrophic factor, was found, which is a member of the nerve growth factor family of proteins.

The RNA-seq analysis revealed that the LeGoiG2-Puro+ empty vector itself significantly impacted many cellular processes, showing its ability to evoke changes in the transcriptome. The number of differentially expressed genes caused by transduction with LeGoiG2-Puro+ and the amplitude of the changes (Supplementary File S1 Figure S6) were so big that perhaps this vector interfered with the TCs induced by some of the TFs used. This is especially true for TF ASCL1 and, to a lesser extent, SOX2. In the case of ASCL1, the impact of the empty vector was of particular functional consequence: the integration of the empty vector LeGoiG2-Pro+ led to increased expression of the TF FOXO3, which is known to compete with ASCL1 for targets [35] and suppress ASCL1-mediated neurogenesis. Thus, based on our transcriptome data, it can be concluded that transgene delivery via the LeGoiG2-Puro+ vector is not suitable when studying ASCL1.

In our study, the number of DEGs and their impact on reprogramming were relatively low in the case of the EE of SOX2. Although another work has shown that SOX2 alone is sufficient to convert mouse fibroblasts into NSCs [6], it was not a potent RF in the case of human fibroblasts in our work.

The EE of NGN2 had the most significant impact on the cell phenotype. Despite the fact that NGN2 and ASCL1 belong to the same family of TFs, supposedly NGN2 is not subject to significant competitive inhibition by FOXO3, which is known to compete with ASCL1. This might be due to the fact that NGN2 and ASCL1 bind slightly different DNA sequence motifs: NGN2 preferentially binds the CAT motif in the E-box, while ASCL1 prefers the CAG motif [27]. Interestingly, in the LF1-NGN2 samples, the expression of the *MASH1* gene, encoding ASCL1, was increased (Figure 4B). Gene ontology analysis revealed in the LF1-NGN2 samples gene expression signatures suggesting a marked activation of pathways related to the functions of potassium and calcium pumps and transport through voltage-gated ion channels, whose activity is characteristic of electrically dependent cells, including neurons. The patterns of gene expression suggested the inhibition of the TF REST1 in the LF1-NGN2 samples (Figure 2D), which is consistent with the literature [36] and is critical for cell fate switch. According to our RNA-seq data analysis, EE of NGN2 led to the activation of both neuron-specific processes, such as the functioning of potassium and calcium pumps and voltage-gated ion channels, which occurs during the formation of mature neurons, and neuron-nonspecific processes, such as the TGFβ signaling cascade. (Having said this, apart from a multitude of functions in other types of cells, TGFβ signaling is also involved in neurogenesis, Figure 2E). Also in LF1-NGN2, the upregulation of *HGMN2* gene transcription was detected, whose product, similar to the aforementioned *HMGB2*, may help to maintain an open chromatin. Further analysis of gene signatures and cellular composition showed that EE of NGN2 ensured the formation of all types of pro-neuronal cells to approximately the same extent, with the exception of ectoderm-like cells (for them, the EE of SOX2 and MSI1 was more impactful). Furthermore, NGN2 made a very significant contribution to the formation of populations of neural stem cells, astrocytic and oligodendrocyte precursors, radial glia, and pericytes.

Surprisingly, the contribution of NGN2 to the generation of populations of mature oligodendrocytes and astrocytes, as suggested based on the bioinformatic analysis, was even larger than that of subpopulations of cells of neuronal subtypes (of these, NGN2 most strongly affected the emergence of glutamatergic and GABAergic neurons). Such results contradict previously published data on the role of NGN2 as a factor of pro-neural differentiation [37]. Furthermore, based on our data, NGN2 activated the transcription of the genes *PAX6*, *HES6*, *SOX11*, *DLL3*, *FABP7*, *SOX9*, *SOX10*, *PAX3*, which are known as “neural stem cell master genes” [38,39,40,41,42,43,44,45,46]. The activation of SOX9 transcription is somewhat counterintuitive, given that in the neural crest, SOX9 mainly suppresses neurogenesis and promotes gliogenic differentiation [26]. Perhaps, the cell fate switch orchestrated by NGN2 involves several steps, starting from a stem-like step before the acquisition of a pro-neuronal phenotype. Also, NGN2 might regulate the expression of a wider array of targets than previously reported. The heterogeneity of the cell populations arising after EE of NGN2 indicates its large-scale influence on a variety of differentiation and transdifferentiation pro-neuronal cascades. Sorting the mixed culture of reprogrammed cells into distinct populations, such as NSCs, neuroblasts, or oligodendrocyte progenitors, or modulating particular pathways via small molecules can help to direct the reprogramming towards the desired target cell types. Alternatively, this factor might be used in combination with additional RFs, which would direct cells to a “narrower” phenotype. Overall, our data confirm the view that NGN2 is capable of generating a plethora of neuronal cell types [17].

The second-best RF in our work, in terms of evoked pro-neural changes in LF1 cells, was MSI1. The expression of MSI1 is not exclusive to the nervous system. It is known as a regulator of embryonic and early postnatal development. It has previously been shown that MSI1 supports the maintenance of NSCs in undifferentiated states via the NOTCH signaling pathway and through translational repression of NUMB expression [47]. Previously, MSI1 was used for direct pro-neural reprogramming [19]. Moreover, the same study reported that cells reprogrammed with the aid of MSI1 had “an intermediate” phenotype between those of radial glia and NSCs. This made them suitable for cell culture, given their proliferative ability; they were also capable of further pro-neuronal differentiation [19]. Many of the DEGs in LF1-MSI1 samples were related to the so-called “general stem cell phenotype” or were common in the transcriptional signatures of radial glia or early NSCs, for example, the *SNAI1* gene [48]. The genes upregulated in LF1-MSI1 do not have a strictly pro-neuronal specialization, and their products are mostly involved in “stemness” of cells. Interestingly, increased transcription of the *HGMN2* gene was observed in the LF1-MSI1 samples. The protein encoded by this gene maintains an open chromatin at the loci of transcribed genes. Next, the level of transcription of some genes involved in the formation of synapses was increased in LF1-MSI1 (such as *MAP1B*, *HSPB1*, *PFN2*), mature neurons (such as the genes *NEFH*, *SLC6A1*, *UCHL1*, *TSPYL2*), and specialized neurons (such as *LMX1B*, *GRIN1*, *GLUL*, and *SLC6A1*). However, the presence of some phenotypic markers of “late neurons” or “specialized neurons” in the absence of others should not be used to support the claim that the cells indeed obtained their functional characteristics, especially given the absence of the corresponding visible morphological changes.

In agreement with previously published data, we found a slightly increased transcriptional activity of “astrocytic genes” associated with EE of MSI1 [18,20]. There was also an elevated transcription of a number of genes of oligodendrocyte progenitors and oligodendrocytes in LF1-MSI1. In all LF1-MSI1 samples, the gene signatures suggested the suppression of the Janus kinase/signal transducer and activator of transcription (JAK-STAT) signaling pathway, the activation of which promotes somatic cell reprogramming [49]. On the other hand, the inhibition of JAK-STAT has been shown to facilitate fibroblast conversion towards other types of cells such as induced myogenic progenitor cells [50] or chondroblasts [51]. Also, the transcriptional signatures in all LF1-MSI1 samples indicated silencing of the signaling via nuclear factor κB (NF-κB), JUN, protein 53 (p53), and interferon regulatory factor 1 (IRF1). p53 is of particular interest. It is called the “guardian of reprogramming” for a reason, as it “oversees” genome integrity maintenance during cell fate conversion at the cost of a diminished efficiency of reprogramming [52]. In several works, suppression of p53 signaling facilitated the pro-neuronal reprogramming [53,54]. In our work, silencing of p53 signaling was observed in LF1-MSI1, LF1-NGN2, and LF1-SOX2, whereas in LF1-LeGOiG2-Puro+, p53 signaling was activated (perhaps in response to lentiviral transduction). Our study also revealed that, depending on the method of RF delivery to the cell, the outcomes might differ. This finding is indirectly confirmed by other previously published data; for example, in the case of iPSC differentiation, surprisingly, the delivery of NGN2 via modified mRNA resulted in mixed populations mainly consisting of neural stem cells, whereas when NGN2 was delivered by the lentiviral system, the majority of the cells were neuronal [55]. In our case, we found that the TCs evoked by the EE of SOX2 alone or ASCL1 alone using the LeGoiG2-Puro+ vector were much less prominent compared to those induced by NGN2 or MSI1. As for ASCL1, the subsequent analysis of TCs revealed that the integration of the empty vector LeGoiG2-Puro+ led to increased expression of the TF FOXO3, which is known to compete with ASCL1 for targets and suppress ASCL1-mediated neurogenesis [35]. Thus, it might be concluded that transgene delivery via the LeGoiG2-Puro+ vector is not suitable when studying ASCL1 and pro-neuronal reprogramming. This conclusion is of direct practical value for all researchers working in this field. As for SOX2, the observed effect might be independent of the impact of the vector, as we found only one work demonstrating that SOX2 alone is sufficient to convert murine fibroblasts into NSCs [6]. The findings of the aforementioned single work were not reproduced in our study in human cells; so, we conclude that indeed it might be not feasible to use SOX2 as a single RF for pro-neural phenotype conversion in human cells. The value of reporting “negative results” is indisputable [55], and these data regarding SOX2-induced TCs in human fibroblasts should be taken into account by those working in the field of direct pro-neural reprogramming.

There are only a few comprehensive transcriptome studies on the early stages of human fibroblast conversion into neuronal cells via the TFs that were used in our work, and to the best of our knowledge, no works with the same experimental design have been performed in human fibroblasts yet. Herdy et al. [26] evaluated the transcriptional changes in human fibroblasts undergoing pro-neuronal reprogramming for 1, 2, 3, 6, 18, and 24 days; NGN2 and ASCL1, simultaneously expressed, were used as RFs. A pathway analysis revealed several pathways affected by the reprogramming: IGF-1 signaling, Rho family GTPase signaling, hypoxia-induced factor 1a (HIF-1a) signaling, integrin signaling, to name but a few [26]. Some of these pathways were affected in our work too, namely, IGF-1 signaling and integrin signaling in the samples LF1-ASCL1 and LF1-MSI1 (but not in LF1-NGN2) and hypoxia-driven pathways in LF1-MSI1 and LF1-NGN2.

The authors also used their data to find, based on RNA-seq pathway analysis, small molecule drugs enhancing pro-neuronal conversion or, supposedly, capable of inducing reprogramming as a single agent. However, they did not determine the cellular composition of the reprogrammed cells using transcriptome data; thus, it remains unknown what the composition was and how similar it is when using TFs or small molecules. Furthermore, the simultaneous use of two RFs, while enhancing the efficiency of reprogramming, made it impossible to reveal the impact of single factors.

The cell phenotype switch induced by NGN2 was accompanied by the formation of a wide range of pro-neuronal cell subpopulations, which included both early stem populations (neural crest cells, radial glia cells, and NSCs), subpopulations of more differentiated cells (immature, mature, and specialized neurons), as well as astroglial and oligodendrocytic cells at varying degrees of maturity. In the case of MSI1, its EE led to the emergence of pro-neuronal cells, but of a much less wide spectrum: subpopulations of early stem cells were predominantly formed, as well as radial glia cells, while more differentiated sub-populations were less represented compared to what observed for LF1-NGN2. Also, MSI1 contributed to the initiation of the formation of marginal astrocytic and oligodendrocytic cell subpopulations.

Consistent with the data from recent works on mouse fibroblasts reprogramming, there was no striking overlap in the transcriptional signatures of different RFs [2,25]. This is not surprising, given the difference in the gene networks regulated by these RFs and the fact that MSI1 is a factor predominantly acting at the level of translation. However, some features of cell phenotype change were common for several RFs used in our study, such as the silencing of the “guardian of reprogramming” p53 signaling during phenotype conversion driven by MSI1, NGN2, or SOX2, or the hypoxia-driven pathways impacted by MSI1 and NGN2. This, while confirming previously published data (pro-neural conversion through different signaling cascades in the case of different RFs [31]), provides a foundation for an informed approach to enhancing RF-driven pro-neural cell conversion by small molecules that target particular pathway(s).

## 4. Materials and Methods

### 4.1. Cultivation of Human Embryonic Pulmonary Fibroblasts

The human embryonic lung fibroblast cell line LF1 was established and characterized by Brown JP et al. [56]. Before lentiviral transduction, LF1 cells were cultured in DMEM cell medium supplemented with 1% GlutaMax and 10% fetal bovine serum (FBS) at 37 °C and 5% CO_2_ in a humidified incubator. After lentiviral transduction, the cells were cultured in (1:1 V/V) DMEM supplemented with 1% GlutaPlus and pyruvate and NeuroBasal Medium supplemented with 1% GlutaMax, in the presence of 3 μM 6-[[2-[[4-(2,4-dichlorophenyl)-5-(5-methyl-1H-imidazol-2-yl)-2-pyrimidinyl]amino]ethyl]amino]-3-pyridinecarbonitrile (CHIR99021), 5 µM N-benzyl-2-(pyrimidine-4-ylamino) thiazol-4-carboxamide (Thiazovivin), 10 µM 7β-Acetoxy-8,13-epoxy-1α, 6β, 9α-trihydroxylabd-14-en-11-one (forskolin), 0.8 ng/mL of noggin, 1% B27, 20 ng/mL of bFGF, and 20 ng/mL of EGF.

### 4.2. Lentiviral Transduction

The cDNAs encoding ASCL1, SOX2, NGN2, and MSI1 were cloned into the LeGOiG2-Puro+ vector to generate the LeGOiG2-Puro+ASCL1, LeGOiG2-Puro+SOX2, LeGOiG2-Puro+NGN2, and LeGOiG2-Puro+MSI1 vectors. The production of replication-incompetent pseudotyped viral particles (PVPs) was carried out in the HEK293T cell line. Briefly, the cells were cultured in DMEM with 10% fetal bovine serum (FBS), 100 U/mL of penicillin, and 100 μg/mL of streptomycin at 37 °C with 5% CO_2_; then, 3 days before transfection, the growth medium was replaced with OPTI-MEM medium without antibiotics; on the day of transfection, the cells reached ~70% confluency. Transfection was performed using the Turbofect reagent according to the manufacturer’s recommendations. The packaging (“helper”) plasmids pLP1, pLP2, and pVSV-G (from Thermo Fisher, Waltham, MA, USA) were used to produce pseudotyped viral particles. The medium with the PVPs was collected and filtered 48 h after transfection. The transduction of the target FL1 cells was performed using the non-concentrated supernatant with PVPs at MOI = 10 in the presence of 10 μg/mL of polybrene. The empty LeGOiG2-Puro+ vector was used for control transduction. Each transduction was performed 3 times, to obtain biological replicates.

### 4.3. RNA Isolation for RNA-Seq

Total RNA was isolated from LF1 cells transduced with LeGOiG2-Puro+ASCL1, LeGOiG2-Puro+SOX2, LeGOiG2-Puro+NGN2, LeGOiG2-Puro+MSI1, and “empty” control vector LeGOiG2-Puro+ and from “mock”-transduced cells. The RNA isolation procedure was carried out on an automatic MagNA Pure Compact nucleic acid isolation device (Roche, Basel, Switzerland) using the MagNA Pure Compact RNA Isolation Kit (Roche, Basel, Switzerland). RNA quantitation was performed using a Qubit 4.0 fluorimeter (Thermo Fisher Scientific, Waltham, MA, USA). The integrity of RNA was assessed using an Agilent 2100 Bioanalyzer (Agilent Technologies, Santa Clara, CA, USA); samples with RNA Integrity Number (RIN) ≥ 9 were used for subsequent analysis.

### 4.4. Reverse-Transcription Real-Time PCR (qRT-PCR)

In parallel with RNA isolation for RNA-seq, total RNA for qRT-PCR was isolated from the same pool of cells using ExtractRNA kit (Evrogen, Moscow, Russia), following the manufacturer’s recommendations. Briefly, RNA isolation was performed by phenol–chloroform extraction followed by precipitation with isopropanol and ethanol. Genomic DNA was removed using the DNase E kit (Evrogen). An additional RNA purification procedure was carried out using the CleanRNA Standard kit (Evrogen) according to the manufacturer’s protocol. RNA quantification was performed by the NanoDrop ND-1000 spectrophotometer. Equal amounts of RNA were used for the reverse-transcription reaction performed using the MMVL RT kit (Evrogen) according to the manufacturer’s protocol. qRT-PCR was carried out in a LightCycler 96 (Roche). The sequences of the primers used were as follows: *GAPDH*, FW 5′-TGCACCACCAACTGCTTAGC-3′, RV 5′-GGCATGGACTGTGGTCATGAG-3′; *ACTB*, FW 5′-CACCATTGGCAATGAGCGGTTC-3′, RV 5′-AGGTCTTTGCGGATGTCCACGT-3′; *MASH1*, FW 5′-CGCGGCCAACAAGAAGATG-3′, RV 5′-CGACGAGTAGGATGAGACCG-3′; *SOX2*, FW 5′-TGCGAGCGCTGCACAT-3′, RV 5′-GCAGCGTGTACTTATCCTTCTTCA-3′; *NEUROG2*, FW 5′-GAGTTTGCAGAGCGGACTGA-3′, RV 5′-GGCATTGTGACGAATCTGGG-3′; and *MSI1*, FW 5′-GGGACTCAGTTGGCAGACTAC-3′, RV 5′-CTGGTCCATGAAAGTGACGAA-3′. The following program was used for amplification: 10 min at 95 °C followed by 40 cycles at 95 °C for 15 s, 57–67 °C for 15 s, 72 °C for 15 s. The specificity of the reaction was confirmed by melting curve analysis. The standard 2−∆∆Ct method was used for data analysis, and two housekeeping genes were used for normalization. All reactions were run at least in duplicates. The 5X qPCRmix-HS SYBR reaction mix (Evrogen) was used for cDNA amplification, as per the manufacturer’s recommendations.

### 4.5. RNA-Sequencing (RNA-Seq)

RNA-seq was carried out at the Center for Genomic Technologies of the Engelhardt Institute of Molecular Biology, Russian Academy of Sciences. Transcriptomic libraries were prepared from 0.2 μg of total RNA using the TruSeq Stranded mRNA Library Prep Kit (Illumina, San Diego, CA, USA) according to the manufacturer’s protocol. The preparation of the libraries included the following steps: (1) enrichment of the mRNA fraction and fragmentation; (2) synthesis of the first and second strands of cDNA; (3) adenylation of the 3′ ends of fragments; (4) ligation of adapters; (5) enrichment of the libraries by PCR. After the enzymatic reactions, cDNA fragments were purified on MagPure A4 XP magnetic particles (Magen, Foshan, China). The concentration of the libraries was measured on a Qubit 4.0 fluorimeter, and the size of the fragments was assessed using automatic capillary electrophoresis on an Agilent 2100 Bioanalyzer. The size of the libraries was estimated as ~260 bp. The libraries were combined equimolarly and diluted to a loading concentration of 1000 pM in RSB buffer with Tween 20 with 1% PhiX control library (Illumina). Sequencing was performed on NextSeq 2000 in paired-end read mode with a length of 51 x 2 nucleons. As a result, at least 20 million sequences per sample were obtained. Each sample had 3 biological replicates. Data availability statement: the raw RNA-seq data are available in the SRA NCBI portal, project PRJNA1182336.

### 4.6. Bioinformatics Analysis

The RNA-seq reads were aligned to the reference transcriptome from Gencode Release 43 (GRCh38.p13) kallisto [57] and STAR [58] and quantified using FeatureCounts [59]. Differential expression analysis was performed using the limma R package 3.44.3 with default thresholds of 4 for fold change and 0.01 for adjusted *p*-value. The selected thresholds were slightly adjusted to ensure a similar distribution of the genes with the most significant changes in expression. Thus, in the case of ASCL1, the threshold was lowered to FC = 1.5 with an adjusted *p*-value of 0.05; for SOX2, the threshold was increased to FC = 2. Enrichment analysis was performed using the ClusterProfiler R package [60]. The activity of the TFs was calculated using decoupleR [61], and the pathways were analyzed using Progeny [62]. The cell subpopulation composition was analyzed using the GBMdeconvoluteR deconv algorithm [63] and the WikiPathways database [64].

### 4.7. Immunocytochemical Staining

For immunocytochemical analysis, the cells were seeded into a 96-well confocal plate (SPL Lifesciences, Pocheon-si, Korea) and cultured as described above. Prior to staining, the cells were fixed in a 4% buffered paraformaldehyde solution. The primary antibodies recognizing beta-III-tubulin (cat# T2200, SIGMA, St. Louis, MO, USA) and nestin (cat# MAB1259, RD System, Minneapolis, MN, USA) were used at a dilution of 1–5 μg/mL. The secondary antibodies goat anti-mouse (H + L) and goat anti-rabbit (H + L) labeled with Alexa Fluor 647, cat# A21235 and A20991 (Invitrogen, Waltham, MA, USA), respectively, were used at dilution of 1:400. The cell nuclei were counterstained with Hoechst (1 μg/mL). The immunocytochemical staining images were obtained using a Nikon A1 scanning laser confocal microscope (Nikon, Minato City, Tokyo, Japan) and analyzed in NIS Elements software version 5.12.03.

## Data Availability

Data availability statement: raw RNASeq data are available in the SRA NCBI portal, project PRJNA1182336.

## Supplementary Materials

The following supporting information can be downloaded at: https://www.mdpi.com/article/10.3390/ijms252212385/s1.

## Abbreviations

BDNF	Brain-derived neurotrophic factor
bHLH	Basic helix–loop–helix structure
CNS	Central nervous system
DCX	Doublecortin
DEGs	Differentially expressed genes
DNA	Deoxyribonucleic acid
EE	Ectopic expression
EGF	Epidermal growth factor
ESCs	Embryonic stem cells
FGF2 (bFGF)	Fibroblast growth factor 2
FOXO3	Forkhead box O3
GABA	Gamma-aminobutyric acid
GFAP	Glial fibrillary acidic protein
NGN2	Neurogenin-2
GSEA	Gene set enrichment analysis
HIF-1a	Hypoxia-induced factor 1a
HSPA2	Heat shock protein family A
IGF-1	Insulin-like growth fractor-1
iPSCs	Induced pluripotent stem cells
JAK	Janus kinase
Mash1 (Ascl1)	Achaete-scute homolog 1
MBD2	Methyl-CpG-binding domain protein 2
Myt1l	Myelin transcription factor 1-like
NeuroD	Neurogenic differentiation
NPCs	Neural progenitor cells
NSCs	Neural stem cells
Olig2	Oligodendrocyte transcription factor 2
OP	Oligodendrocyte precursor
Pax6	Paired box protein Pax-6, also known as aniridia type II protein (AN2) or oculorhombin
PCA	Principal component analysis
POU3F3	POU class 3 homeobox 3
qRT-PCR	Real-time polymerase chain reaction with reverse transcription
REST	RE1-silencing transcription factor
RFs	Regulatory factors
RNA	Ribonucleic acid
SOX2	SRY-box transcription factor 2
TCs	Transcriptional changes
TFs	Transcriptional factor
TTBK1	Tau tubulin kinase 1
Wnt	Wingless-type MMTV integration site
