# Supplementary material for: A Snapshot of Early Transcriptional Changes Accompanying the Pro-Neural Phenotype Switch by NGN2, ASCL1, SOX2, and MSI1 in Human Fibroblasts: An RNA-Seq Study
Source: Int J Mol Sci. 2024 Nov 18;25(22):12385. doi: 10.3390/ijms252212385 (PMC11594342; doi:10.3390/ijms252212385)
Supplement: Supplementary file 1 [file ijms-25-12385-s001.zip › ijms-3269507-supplementary/Supplementary files/Supplementary 1.pdf]

Supplementary 1

Supplementary figure 1

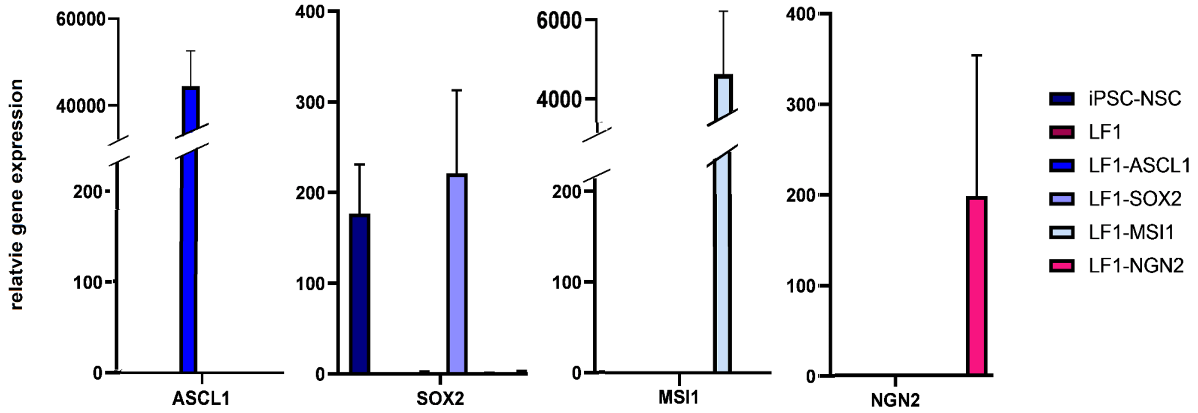

Figure S1. Ectopic expression of ASCL1, SOX2, MSI1 and NGN2 RFs. (a) Gene expression levels on day 5 after transduction, fold change relative to LeGOiG2-Puro+. LF1 - “mock” transduced cells, LF1-LeGOiG2-Puro+ - cells transduced with control vector LeGOiG2-Puro+, LF1-ASCL1, LF1-SOX2, LF1-MSI1 and LF1-NGN2 - cells transduced with LeGOiG2-Puro+ASCL1, LeGOiG2-Puro+SOX2, LeGOiG2-Puro+MSI1, LeGOiG2-Puro+NGN2, correspondingly.

Supplementary figure 2

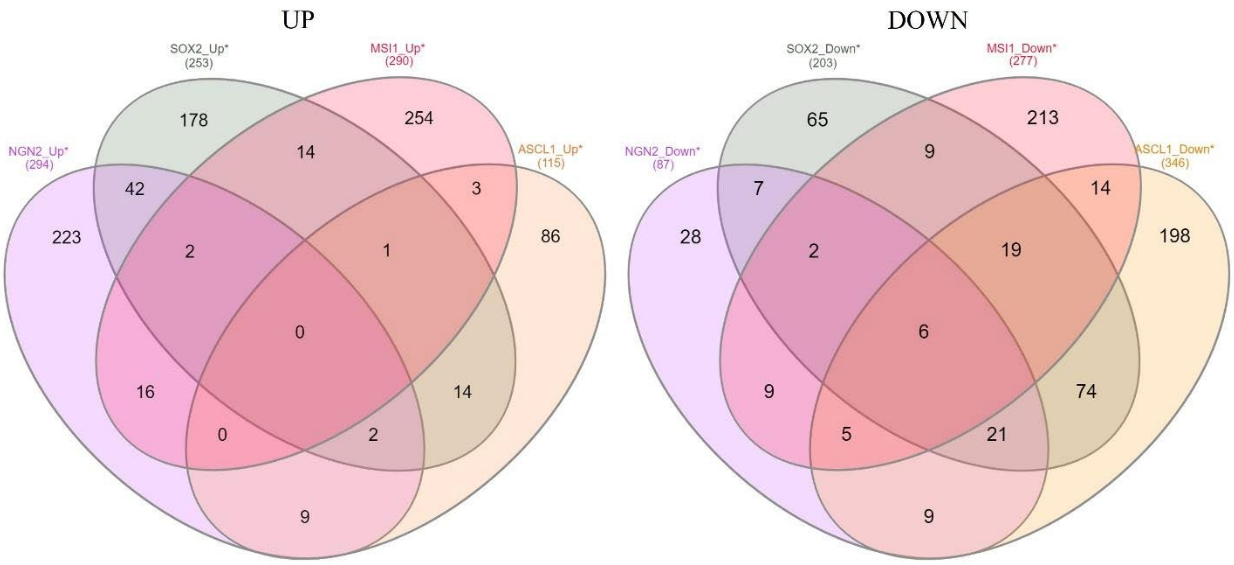

Common DEGs across LF1-NGN2, LF1-MSI1, LF1-SOX2 and LF1-ASCL1 samples.

Venn diagrams of the DEGs that were significantly upregulated and downregulated in all samples but not in LF1-LeGoiG2-Puro+

Supplementary figure 3

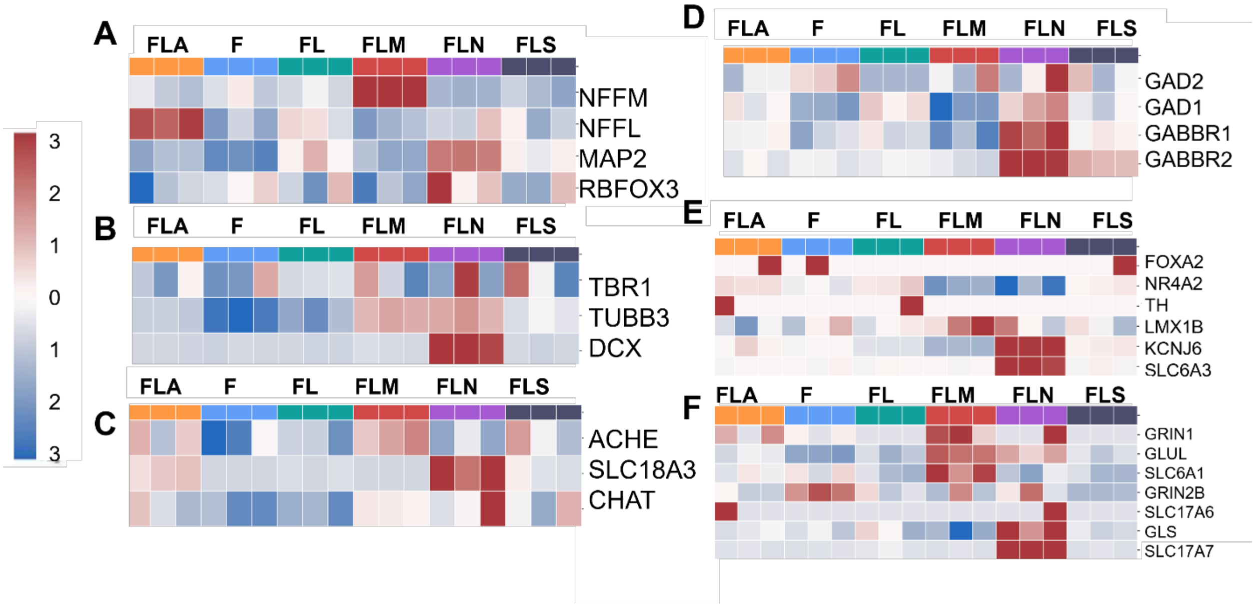

Profile of cell subtypes in the samples based on gene expression analysis. Heatmap showing enrichment of samples with DEGs of mature (A) and young neurons (B), cholinergic (C) and GABA-ergic neurons (D). F – control fibroblasts LF1, FL – LF-LeGoiG2-Puro+, FLA - LF-LeGoiG2-Puro+-ASCL1, FLM - LF-LeGoiG2-Puro+-MSI1, FLN - LF-LeGoiG2-Puro+-NGN2, FLS - LF-LeGoiG2-Puro+-SOX2. Blue and red color scale represents median-scaled change in gene expression or cell population enrichment (red - up, blue - down).

Supplementary figure 4

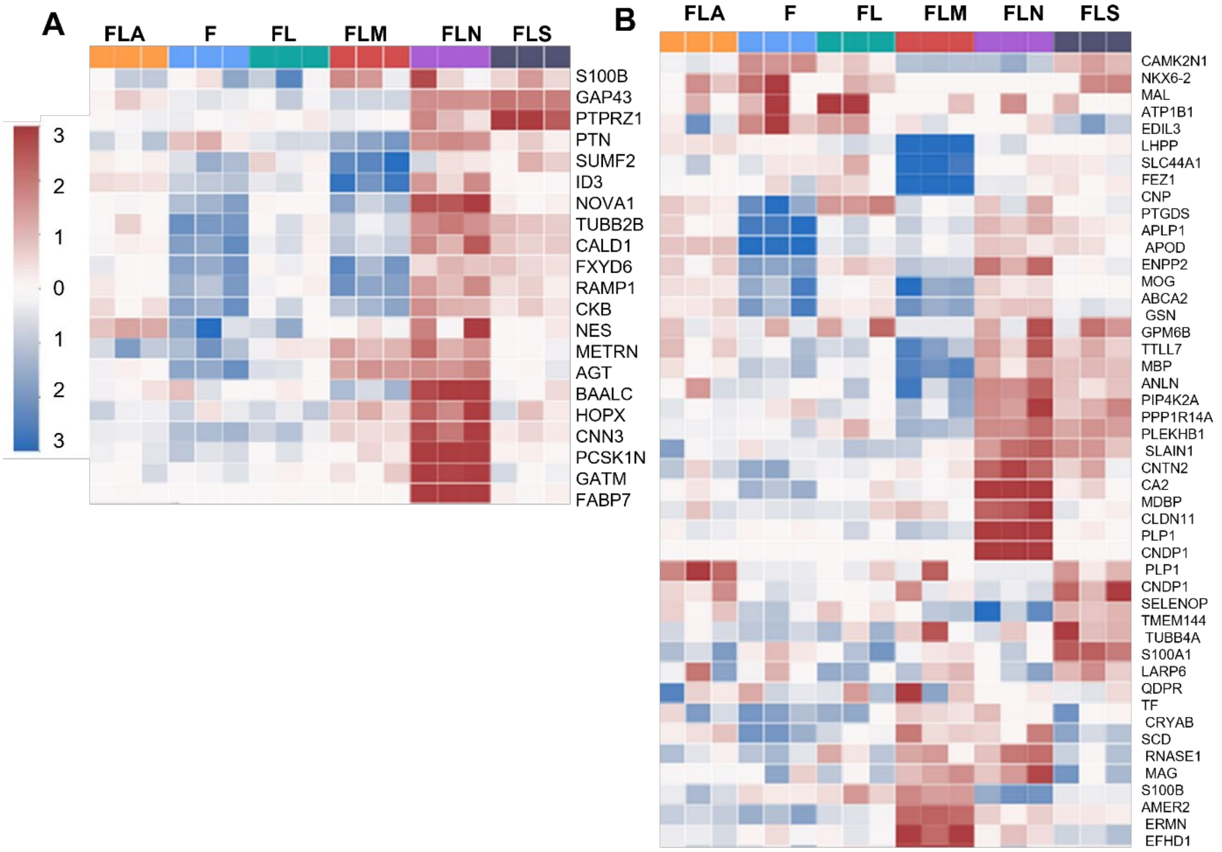

Profile of cell subtypes in the samples based on gene expression analysis. Heatmap showing enrichment of samples with DEGs of astrocyte progenitors (A) and oligodendrocytes (B). F – control fibroblasts LF1, FL – LF-LeGoiG2-Puro+, FLA - LF-LeGoiG2-Puro+-ASCL1, FLM - LF-LeGoiG2-Puro+-MSI1, FLN - LF-LeGoiG2-Puro+-NGN2, FLS - LF-LeGoiG2-Puro+-SOX2. Blue and red color scale represents median-scaled change in gene expression or cell population enrichment (red - up, blue - down).

Supplementary figure 5

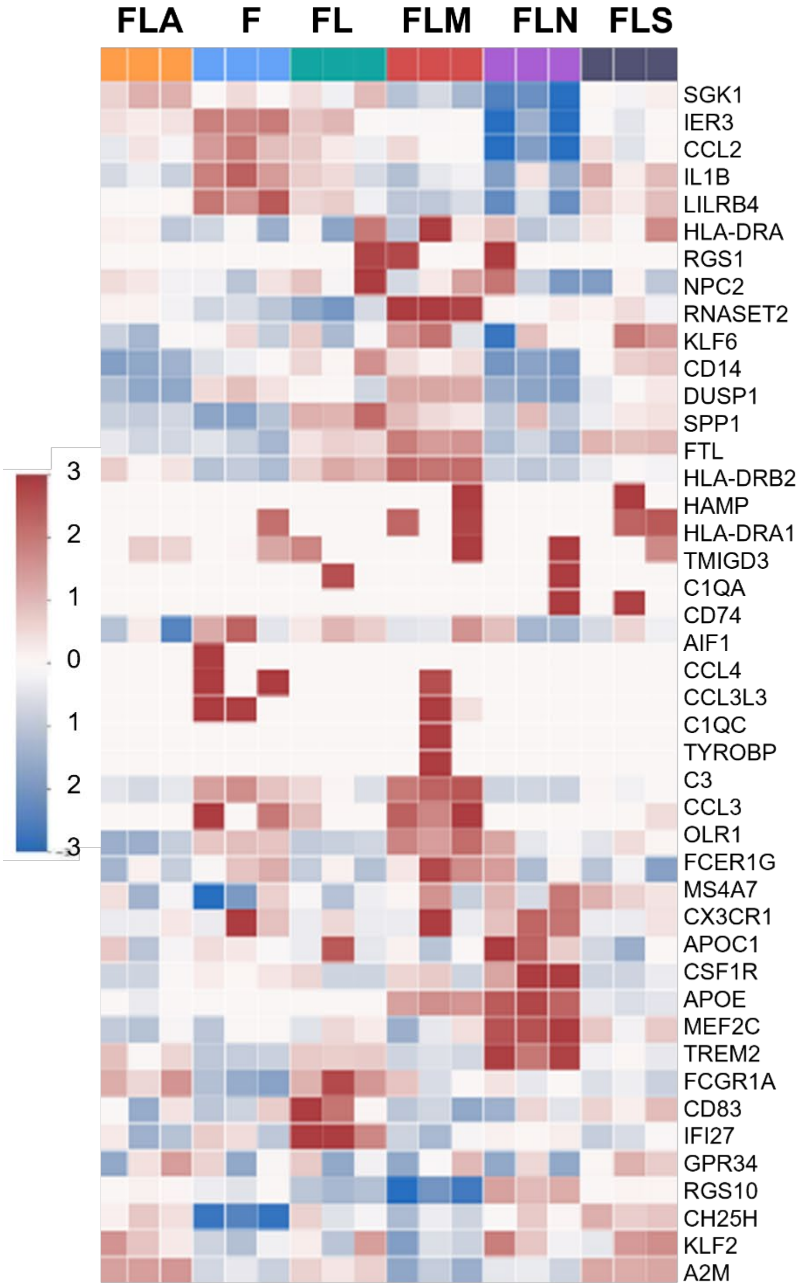

Profile of cell subtypes in the samples based on gene expression analysis. Heatmap showing enrichment of samples with DEGs of microglia. F – control fibroblasts LF1, FL – LF-LeGoiG2-Puro+, FLA - LF-LeGoiG2-Puro+-ASCL1, FLM - LF-LeGoiG2-Puro+-MSI1, FLN - LF-LeGoiG2-Puro+-NGN2, FLS - LF-LeGoiG2-Puro+-SOX2. Blue and red color scale represents median-scaled change in gene expression or cell population enrichment (red - up, blue - down).

Supplementary figure 6

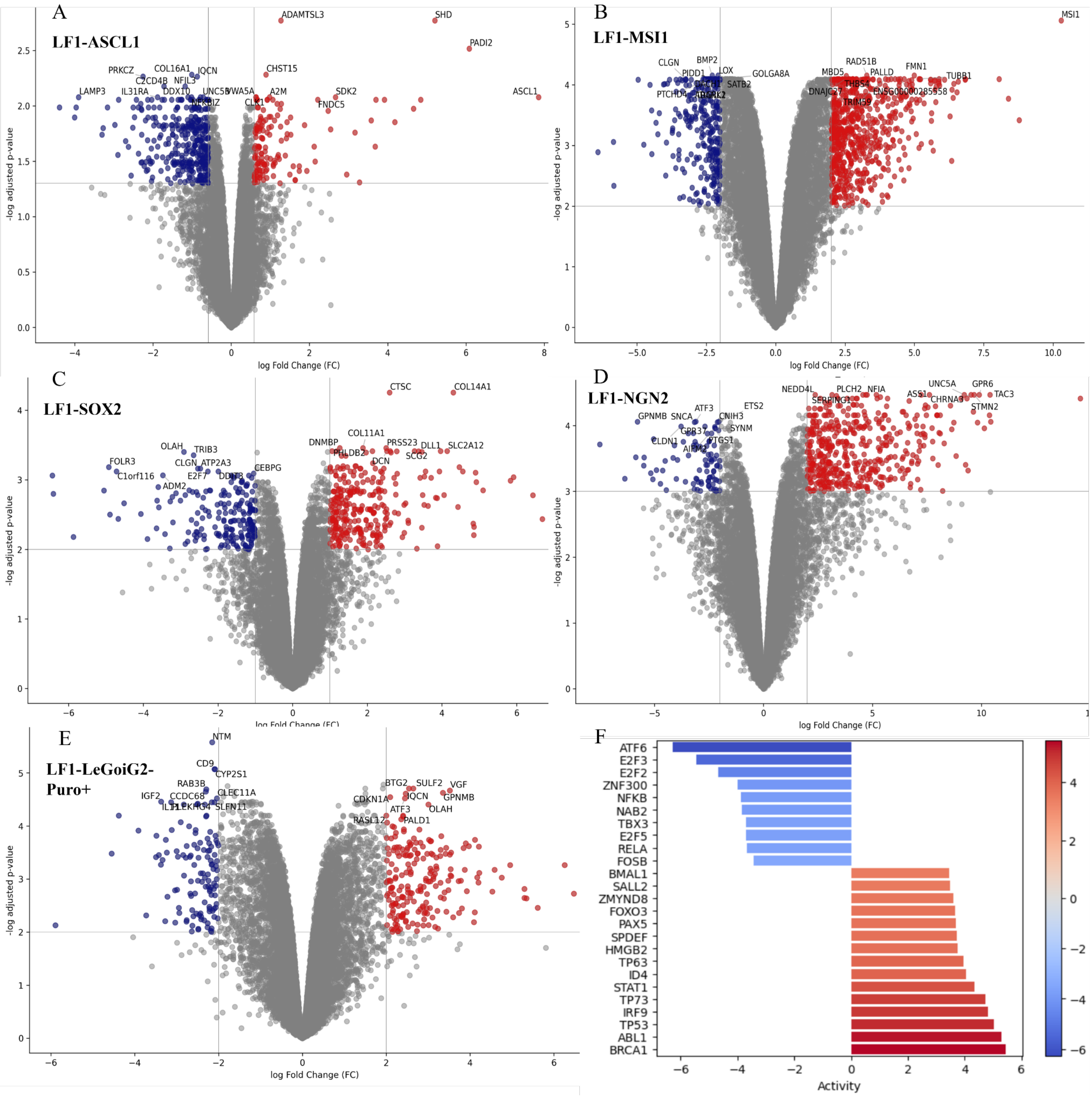

Volcano plots of DEGs. Data for LF1-ASCL1 (A), LF1-MSI1 (B), LF1-SOX2 (C), LF1-NGN2 (D) and LF1-LeGoiG2-Puro+ (E) are presented. (F) The most highly affected transcription factors are shown based on the profiles of their target DEGs expression in LF1-LeGoiG2-Puro+.
